# Swedish version of the Pregnancy Physical Activity Questionnaire (PPAQ-SWE), translation and cultural adaptation

**DOI:** 10.1186/s13104-025-07135-0

**Published:** 2025-02-07

**Authors:** Annika Svahn Ekdahl, Viana Mikkelsen, Heléna Frenell, Qarin Lood, Theresa Westgård, Joanne M. Fuller, Andrea Mikkelsen, Annelie Gutke

**Affiliations:** 1 Department of Health and Rehabilitation, Institute of Neuroscience and Physiology, The Sahlgrenska Academy, University of Gothenburg, Box 455, Gothenburg, SE-408 30 Sweden; 2 School of Nursing and Midwifery, Faculty of Health Science, La Trobe University, Melbourne, Australia; 3 Administration for the Elderly, Nursing and Care, Department of Quality and Development, The City of Gothenburg, Gothenburg, Sweden; 4 The Gothenburg University Centre for Ageing and Health (AgeCap), Gothenburg, Sweden; 5 Centre for Person‑Centred Care (GPCC), University of Gothenburg, Gothenburg, Sweden; 6 Department of Psychiatry and Neurochemistry, Institute of Neuroscience and Physiology, The Sahlgrenska Academy, University of Gothenburg, Gothenburg, Sweden; 7 Institute of Health and Care Sciences, The Sahlgrenska Academy, University of Gothenburg, Gothenburg, Sweden; 8 Department of Clinical Neuroscience/Ophthalmology, Institute of Neuroscience and Physiology, The Sahlgrenska Academy, University of Gothenburg, Gothenburg, Sweden; 9 Department of Internal Medicine and Clinical Nutrition, Sahlgrenska Academy, University of Gothenburg, Gothenburg, Sweden; 10 Research and Development Primary Health Care, Gothenburg and Södra Bohuslän, Region Västra Götaland, Sweden

**Keywords:** Obstetrics, Physical activity, Physiotherapy, Pregnancy, Translation, Women’s health

## Abstract

**Objective:**

Physical activity has well-known health benefits in all stages of life and may also reduce the risk for pregnancy-related complications, but many pregnant women do not reach the recommended activity levels. Tailored advice is often provided by healthcare professionals to promote physical activity during pregnancy. To provide such advice, assessment of the actual level of physical activity is necessary, but there are currently no standardized methods for this. The Pregnancy Physical Activity Questionnaire (PPAQ) is a self-administered instrument, designed to determine physical activity during pregnancy but a Swedish version of the questionnaire is not yet available.

**Result:**

The PPAQ was translated and culturally adapted into a Swedish version, i.e., PPAQ-SWE according to the process described by Beaton et al. Several minor linguistic adjustments were needed, that mainly concerned semantic and cultural adaptations, such as adding, removing, or replacing words to match Swedish cultural aspects. For instance, “gestational week” was used instead of “trimester,” and “older person” replaced “older adult.” The reference to a “1-gallon milk jug” was replaced with “3–4 liters of milk” to fit the metric system used in Sweden. Two questions regarding bicycling, a common form of transportation in Sweden, and one free-text comment option was added.

## Introduction

The purpose of this work was to translate and culturally adapt the Pregnancy Physical Activity Questionnaire (PPAQ), a questionnaire used for assessing physical activity level in pregnant women, into Swedish.

Physical activity provides health benefits throughout all stages of life, including pregnancy. The risk of pregnancy-related complications such as high blood pressure, diabetes, overweight/obesity, and depressive symptoms may be reduced if the woman is physically active [1–4]. Pregnant women are advised to engage in 150–300 min of moderate-intensity or 75–150 min of high-intensity physical activity per week [4, 5], yet many do not meet these recommendations [6, 7]. Barriers for physical activity may include pregnancy symptoms, fatigue, lumbopelvic pain, time constraints, and concerns about fetal safety, while social support, and professional guidance can help facilitate activity [8–12].

Pregnant women often seek advice from healthcare professionals due to pregnancy-related complaints that hinders physical activity [13]. Addressing knowledge gaps and offering tailored advice are key to improving adherence to recommended activity levels [14, 15]. To provide tailored advice, assessment of the individual’s actual level of physical activity is required, as structured and supervised exercise has been shown to improve pregnant women’s motivation and exercise self-efficacy [16]. Integrating physical activity assessment and education into routine prenatal care, may help bridge knowledge gaps and promote long-term maternal and fetal health [14].

Since no standardized method for measuring physical activity in pregnancy exists, current assessments rely on a combination of objective and subjective measures [17, 18]. The PPAQ [19] is a self-administered questionnaire designed to assess pregnant women’s physical activity levels during an average day or week. It consists of 36 items: three background items, and 33 items focused on time spent on various activities within four domains: household/caregiving activities, work-related activities, sports/exercise, and transportation. The time spent on each activity is converted into a Metabolic Equivalent of Task (MET), indicating physical activity energy expenditure levels relative resting expenditure. According to Chasan-Taber et al. [19], MET values calculated from PPAQ classify activities as sedentary (< 1.5 METs), light (1.5–3.0 METs), moderate (3.0–6.0 METs), or vigorous (> 6.0 METs). The time spent on each activity is multiplied by its intensity to calculate the average weekly- or daily energy expenditure (MET-hours/week or per day). Responses can be summed to classify total activity, intensity, and/or for each activity domain [19].

The PPAQ from 2004 has been updated and validated by the original author in 2022 [20]. The results show that the updated questionnaire may detect changes in physical activity and is a valid and reliable instrument for measuring physical activity, at different intensities, during pregnancy. Spearman’s correlation coefficient between PPAQ and accelerometer for total physical activity level (MET-hours/day) range from 0.37 to 0.44 during the three trimesters of pregnancy. For reproducibility, the intra class correlation coefficient (ICC) ranges from 0.55 to 0.83 for total activity [20]. The PPAQ is a recommended instrument for assessing physical activity in pregnancy [21], and it has been translated into several languages, but a Swedish version is currently unavailable.

## Method

The translation and cultural adaptation of PPAQ into a Swedish version were conducted according to Beaton et al. [22]. A written approval from the original author was obtained in March 2022.

In the first step, four female translators performed a forward translation from English to Swedish. Two physiotherapy students in collaboration (VM, HF), an occupational therapist (QL) and a dietician (AM) translated individually. The students were native Swedish speakers with strong English skills and medical vocabulary. The other two translators (QL, AM) also had clinical experience and excellent English skills, with one being a native Swedish speaker (QL). In the second step, all four translators compared and compiled their translations to reach a consensus on the Swedish version.

At the third step, two female, native English speakers, an occupational therapist (TW), and a pharmacist with public health training (JMF), conducted the back-translation independently. To avoid bias, they did not have access to the original questionnaire or had prior experience of the PPAQ. They thereafter discussed the individual translations and compared them to the original English version, resulting in a joint back-translation.

The fourth step included an expert committee meeting with all participating translators and two independent researchers (ASE, AG, both female physiotherapists). During this meeting, the compiled translated versions were discussed and assessed for face- and content validity. Difficulties in translation and cultural adaptation were evaluated, focusing on making the questionnaire relevant for the target group. The expert committee meeting resulted in a preliminary Swedish version of the PPAQ, PPAQ-SWE.

In step five, an additional evaluation of face- and content validity for the PPAQ-SWE was conducted digitally by six female physiotherapists at the University of Gothenburg, all with vast clinical experience and prior personal experiences of pregnancy. They received written information about the study and provided consent as they returned the questionnaire. The physiotherapists had no prior knowledge of the PPAQ, answered the questionnaire based on a hypothetical pregnancy and documented any ambiguities. They evaluated their experience of the questionnaire, based on cognitive interviewing methodology [23], and provided written feedback on any difficulties they encountered.

## Results

A Swedish version of the PPAQ is now available. In the process of translation and cultural adaptation, minor semantic and cultural changes were needed, for example adding or removing words and/or replacing words to match cultural aspects. In the original version, the dates were given in the American format month/day/year, and this was changed to the Swedish standard format year/month/day. In Sweden, the current gestational week is more commonly used (both in colloquial language and in the clinical history) than trimester. Therefore, the question about gestational timepoint at the beginning of the questionnaire was modified, i.e. “during the current period of your pregnancy”, instead of “during this trimester”.

To avoid incomplete Swedish sentences in the translation, the word “on” was added so the initial phrase in the instructions for each question section was formulated “…how much time do you usually spend on…”.

The question concerning caring for an older adult was modified, “older adult” was replaced with “older person”, and “taking care of” was changed to “helping” to better reflect the Swedish context. The word “helping” allowed for a broad interpretation of activities included in helping an older person.

The phrasing “sitting down reading” in the original questionnaire was adapted to today’s digital society and cultural context where screens are used more frequently than when the original PPAQ was constructed [19]. This resulted in the addition of devices such as computers, tablets and smartphones to this question in the PPAQ-SWE.

In English, the word “homemaker” is gender-neutral; in Swedish, there is no equivalent word. Therefore, a descriptive phrasing was chosen: “If you mainly work with managing the household…”.

Since the metric system is used in Sweden, the phrase “a 1-gallon milk jug” included in the questions regarding carrying objects was not relevant. Both translators and the expert committee discussed this to find something similar, often used in Swedish everyday life. The original phrase was replaced by “3–4 liters of milk”.

In Sweden, cycling is a common form of transportation also for pregnant women, but this was not included in the original version [19]. Therefore, two questions were added to investigate the time spent on cycling for transportation, on low intensity/electric bicycle (MET = 6.8), or on moderate to high intensity (MET = 10). The MET-values for these two activities were taken from the 2011 Compendium of Physical Activities [24].

A possibility for the participants to write additional information and/or comments was added, which resulted in a total of 39 questions in the Swedish version.

### Validity

The six physiotherapists who completed the preliminary PPAQ-SWE described the questionnaire as easy to understand, with a response time of approximately ten minutes which is similar to the original version [19]. They emphasized the importance of clearly marking the response categories as they vary from daily to weekly, to avoid misinterpretation of the time spent on each activity. The results from this face-, and content validity testing did not result in any major revisions of PPAQ-SWE, only minor semantic changes were suggested to enhance comprehension.

## Discussion

The PPAQ-SWE is a suitable instrument that captures physical activities on different intensity levels that pregnant women might do in their everyday life. Pregnant or postpartum women are encouraged to meet the same recommendations for physical activity as other healthy adults [5], but their physical activity often tends to decrease as pregnancy proceeds [25] and to remain on a low level postpartum [26]. Even if women postpartum report at least 150 min/week, their physical activities are mostly on a low intensity level, and moderate to high intensity activities are performed in too short bouts to achieve positive health benefits according to the recommendations [27]. Therefore, it is important to assess both the type of activity and at what intensity the activity is performed to be able to give individually tailored advice to pregnant women regarding physical activity and to reduce sedentary time.

The relevance of the question about helping an older person was discussed, and whether it should be removed from the questionnaire. While it was removed in the Danish version [28, 29], the Swedish authors chose to retain it in the PPAQ-SWE as it reflects activities that pregnant women may engage in, ranging from higher-intensity tasks (e.g., personal hygiene, cleaning, gardening) to lower-intensity activities (e.g., paying bills, attending appointments). Informal caregiving is more common among women than men and can negatively affect their mental and physical health, especially as they may care for children, aging parents, and relatives simultaneously [30]. Since the specifics of caregiving and/or helping are not detailed in the question, the MET-value assigned would be an estimate.

After the translation process was concluded, an updated version of the original PPAQ, including a new validation [20], was published and the preliminary Swedish version was thus revised accordingly. The updated version of PPAQ covers “screen-time” as added in the Swedish version. A discussion arose regarding three questions, all of which include sitting activities on low intensity, and if they could be combined into one. However, the decision was to keep three separate questions to avoid the risk for underestimation of sedentary time if the individual questions were merged into one.

In Sweden, both regular and electric bicycles are commonly used for transportation. Since cycling wasn’t included in the original PPAQ [19, 20], we identified a need to add it to the PPAQ-SWE. Cycling is also a common form of exercise, whether on a stationary, road, or mountain bike, raising concerns about potential misinterpretation as both exercise and transportation. The authors agreed that cycling for exercise could be recorded separately, as the questionnaire allows women to add any activity, other than those listed, for fun or exercise. Adding cycling in the PPAQ-SWE corresponds with for example the Danish [28, 29], Japanese [31], Polish [32], and Vietnamese [33] versions, which recognizes cycling as a common form of transportation.

## Limitations

Face validity was assessed by six physiotherapists, which could be a limitation as they might have answered the questionnaire based on their professional knowledge, rather than any personal pregnancy experiences. Hence, their responses may differ from the actual target group. In Denmark, with societal and cultural comparable conditions to Sweden, PPAQ has been translated by two separate research groups showing suitability for pregnant women [28, 29]. The Danish version showed acceptable validity, as it was easy to complete and included relevant activities, test re-test reliability (ICC for total activity 0.79) and internal consistency (Cronbach’s alpha for the total activity scale 0.70) was acceptable [29]. Based on the cultural similarities with Denmark and the validation of the Danish instrument, we decided that no separate testing was needed for the PPAQ-SWE.

## Conclusion

The PPAQ-SWE is now available for use in clinical work and/or research among healthcare professionals who provide clinical services for pregnant women. The questionnaire is easy to use and can serve as a basis for discussion to offer tailored advice based on the individual’s physical activity level in any stage of pregnancy.

## Data Availability

Data sharing is not applicable to this article as no datasets were generated or analyzed during the current study.

## Abbreviations

ICC: Intra Class Correlation
MET: Metabolic Equivalent of Task
PPAQ: Pregnancy Physical Activity Questionnaire (original)
PPAQ-SWE: Swedish version of the Pregnancy Physical Activity Questionnaire
WHO: World Health Organization
